# Opioid Education and Nasal Naloxone Rescue Kits in the Emergency Department

**DOI:** 10.5811/westjem.2015.2.24909

**Published:** 2015-04-01

**Authors:** Kristin Dwyer, Alexander Y. Walley, Breanne K. Langlois, Patricia M. Mitchell, Kerrie P. Nelson, John Cromwell, Edward Bernstein

**Affiliations:** * Boston University School of Medicine, Boston Medical Center, Department of Emergency Medicine, Boston, Massachusetts; † Boston University School of Medicine, Boston Medical Center, Department of Medicine, Boston, Massachusetts; ‡ Boston University School of Public Health, Department of Biostatistics, Boston, Massachusetts; § Boston University School of Public Health, Department of Community Health Sciences, Boston, Massachusetts

## Abstract

**Introduction:**

Emergency departments (EDs) may be high-yield venues to address opioid deaths with education on both overdose prevention and appropriate actions in a witnessed overdose. In addition, the ED has the potential to equip patients with nasal naloxone kits as part of this effort. We evaluated the feasibility of an ED-based overdose prevention program and described the overdose risk knowledge, opioid use, overdoses, and overdose responses among participants who received overdose education and naloxone rescue kits (OEN) and participants who received overdose education only (OE).

**Methods:**

Program participants were surveyed by telephone after their ED visit about their substance use, overdose risk knowledge, history of witnessed and personal overdoses, and actions in a witnessed overdose including use of naloxone.

**Results:**

A total of 415 ED patients received OE or OEN between January 1, 2011 and February 28, 2012. Among those, 51 (12%) completed the survey; 37 (73%) of those received a naloxone kit, and 14 (27%) received OE only. Past 30-day opioid use was reported by 35% OEN and 36% OE, and an overdose was reported by 19% OEN and 29% OE. Among 53% (27/51) of participants who witnessed another individual experiencing an overdose, 95% OEN and 88% OE stayed with victim, 74% OEN and 38% OE called 911, 26% OEN and 25% OE performed rescue breathing, and 32% OEN (n=6) used a naloxone kit to reverse the overdose. We did not detect statistically significant differences between OEN and OE-only groups in opioid use, overdose or response to a witnessed overdose.

**Conclusion:**

This is the first study to demonstrate the feasibility of ED-based opioid overdose prevention education and naloxone distribution to trained laypersons, patients and their social network. The program reached a high-risk population that commonly witnessed overdoses and that called for help and used naloxone, when available, to rescue people. While the study was retrospective with a low response rate, it provides preliminary data for larger, prospective studies of ED-based overdose prevention programs.

## INTRODUCTION

In the United States, deaths from prescription opioid overdose increased from 4,041 in 1999 to 16,651 in 2010.^1^ In 2011, an estimated 420,040 emergency department (ED) visits were prescription opioid-related and 258,482 were heroin-related.^2^ The Office of National Drug Control Policy recognizes a “window of opportunity to intervene by calling 911, giving rescue breathing and by the administration of naloxone by a trained lay person.”^3^ Overdose education and naloxone distribution (OEN) programs educate those at risk for opioid overdose or those likely to witness an overdose to prevent, recognize and respond. As of 2010, OEN programs had been implemented in 188 communities nationwide to address this epidemic. Traditionally these programs were located in needle syringe programs. Over 53,032 individuals were trained in OEN from 1996 through 2010, resulting in 10,171 overdoses reversed with naloxone.^4^ Previous studies have found implementation of OEN programs is associated with reduced opioid overdose death rates,^5^–^9^ and is cost-effective among heroin users.^10^ Through 2014, 25 U.S. states and the District of Columbia have amended their laws to allow physicians to prescribe and dispense the drug and to allow the lay public to administer naloxone without legal consequence.^11^ Given the frequency of opioid-related visits, the ED may be a high-yield venue for overdose prevention interventions. To date, no published studies have described an ED-based OEN program that includes naloxone distribution.

Our objectives were to evaluate the feasibility of an ED-based overdose prevention and intervention program, and describe the overdose risk knowledge, opioid use, overdose, and overdose response actions among ED patients who received overdose education only (OE) or OEN in this observational study.

## METHODS

### Study Design

We conducted a survey of OE and OEN patients who had been seen in our ED between January 1, 2011 and February 28, 2012. Trained research assistants (RAs) interviewed participants by telephone between March 1, 2012 and October 31, 2012. Data entry, abstraction and analysis were performed by data analysts. The local institutional review board approved this study.

### Study Setting and Population

This study was conducted at an academic, urban, Level I trauma center with racially and ethnically diverse patients. All patients who spoke English and were seen by our ED-based licensed alcohol and drug counselors (LADC) for OE or OEN were eligible for inclusion.

### Study Protocol

Initially started in 1993 with funding from the Substance Abuse Mental Health Services Administration, Project ASSERT (PA) has been funded by the hospital since 1997, with a staff of LADCs that collaborates with ED providers to offer substance-abuse screening, brief intervention and referrals to substance use disorder treatment.^12^ In 2009, PA implemented an overdose education program in accordance with the Massachusetts Department of Public Health (MDPH) overdose prevention pilot program for patients at risk for opioid overdose.^5^ The LADCs dispense free nasal naloxone rescue kits to at-risk ED patients under a standing order from the MDPH medical director. OEN takes approximately five minutes, and while the kits cost $55 for two atomized 2mg naloxone vials, they are currently state funded.

Receipt of a naloxone kit was not randomized but was primarily dependent on trained staff availability and patient preference during the ED visit. OE and OEN patients seen by PA were educated about overdose risks and how to recognize and respond to a witnessed overdose by calling 911, delivering rescue breaths, and staying with the person until EMS arrives. A list of ED patients seen by PA who received OE or OEN was generated from ED electronic records and their phone numbers were extracted from the billing database. RAs contacted subjects from this list, obtained informed consent, and administered the survey. RAs attempted to make contact up to 10 times before excluding the subject. We excluded participants with disconnected or inaccurate phone numbers.

### Data collection and Measurements

Survey questions included: demographics, overdose education and naloxone history, personal overdose history, witnessed overdose history, past 30-day substance use, and overdose risk knowledge retention (Appendix 1).

### Data Analysis

We present descriptive data from our study and comparisons between OE and OEN groups among those patients who responded to the survey. We defined opioid use as any self-reported opioid use in the past 30 days. Opioid overdose was defined as any self-reported overdose since the ED index visit. To assess participants’ overdose response behavior we asked participants about the following: 1) calling 911; 2) rescue breathing; 3) administering naloxone; and 4) staying with the victim. We used chi-square tests (Fisher’s exact when appropriate) to compare these groups. All analyses were conducted in SAS v. 9.3.

## RESULTS

There were 415 patients seen by PA during the study period; 359 received OE only and 56 received OEN. Among the 415, 12% (51/415) completed surveys; 4.6% (19/415) were reached but did not complete surveys; 38% (156/415) had wrong or disconnected phone numbers; 35% (147/415) were not reached after 10 attempts; 10% (40/415) had no phone number; 0.5% (2/415) were reported as deceased from an overdose. The median time between ED index visit and survey completion was 12 months for OE only (range: 8–17 months), and 11 months for OEN (range: 5–19 months).

Among the 51 patients who completed the survey, 73% (37/51) had received naloxone (Figure). Among these, 76% (28/37) of respondents received a kit from the ED, and 24% (9/37) received their kit elsewhere, such as a detox facility. Past 30-day opioid use was reported by 35% of those surveyed and 22% self-reported surviving an overdose. Among the 27 participants who witnessed an overdose, 63% called 911, 22% performed rescue breathing, 22% used a naloxone kit to reverse the overdose, and 93% stayed with victim. We detected no significant differences in behavior in a witnessed overdose between the OEN and OE-only groups. In the OEN group, 16% (6/37) reported using their kit to successfully reverse a witnessed overdose, one person reported their kit was used by someone else to rescue an overdose victim, and 54% (20/37) still had their kits in their possession.

## DISCUSSION

This brief report describes the implementation of an opioid harm reduction public health intervention in the ED setting. The participants represent a high-risk population; between their ED visit and study interview, more than one fifth reported a non-fatal overdose, over half witnessed an overdose and there were two overdose deaths reported. In this small sample, we did not detect statistically significant differences between OE-only and OEN behavior in a witnessed overdose, reported opioid use or overdose rates. Almost one third (6/19) of the OEN group who witnessed an overdose used naloxone to rescue someone and more than half of the OEN group still had a naloxone rescue kit.

The ED provides a promising opportunity for risk-reduction measures for opioid overdose, including naloxone rescue kits. Although no significant differences were detected in overdose response behaviors, the group with naloxone rescue kits did have higher rates of calling 911, administering naloxone and staying with the victim until help arrived. While a dedicated substance use service, such as PA, is not available in most EDs, ED providers, including social workers, can offer OE or OEN in the ED without PA. There are useful tools for setting up an OE or OEN program,^13^ including a prescription for naloxone with OE information, which can be found at prescribetoprevent.org (Appendix 2). An increasing number of outpatient pharmacies stock nasal naloxone. Thus, ED providers can work with their hospital outpatient pharmacy to stock kits. In September 2013, as a result of this pilot project, the hospital adopted a policy to make OEN accessible to all high-risk ED patients prior to discharge, not only through PA, but also through the inpatient and outpatient pharmacies.

## LIMITATIONS

Our follow-up interview enrollment was low as we were limited to hospital billing data for participant phone numbers and many numbers were incorrect or no longer in service. However, we were able to reach 50% of the OEN group. Patients were exposed to OE in the ED, but they may also have received OE at other venues. The decision to provide a nasal naloxone kit was not randomized, and therefore the sampling was subject to selection bias and may not be generalizable. Because this study was a survey, responses to questions may have been subject to social desirability and recall bias. The chart abstractors were not blinded to the study hypothesis. As we did not survey patients without exposure to overdose education, we do not have a non-OE control group. To pursue these initial findings further, larger prospective studies are warranted as OEN programs are implemented in EDs.

## CONCLUSION

The ED provides a promising opportunity for opioid overdose harm reduction measures and naloxone rescue kit distribution to laypersons and bystanders encountered during an ED visit. This is the first description and evaluation of an ED-based nasal naloxone rescue kit program. The program reached a high-risk population that commonly witnessed overdoses, called for help and used naloxone to rescue people, when available. This study provides useful information for planning larger studies and programs to further evaluate implementation, benefits and harms of OEN in EDs.

## Supplementary Information





## Figures and Tables

**Figure:**
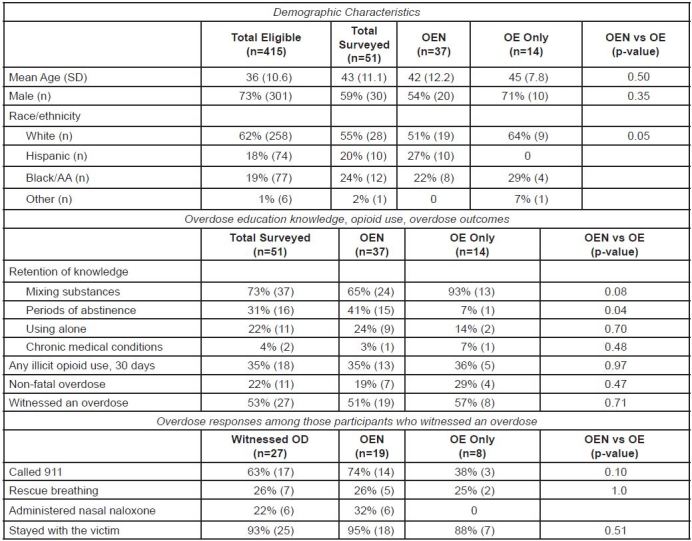
Opioid education and nasal naloxone rescue kits in the emergency department. *AA*, African American; *OEN,* overdose education and nasal naloxone rescue kit*; OE,* overdose education only*; OD,* overdose
