# Supplementary material for: Opioid Education and Nasal Naloxone Rescue Kits in the Emergency Department
Source: West J Emerg Med. 2015 Apr 1;16(3):381–4. doi: 10.5811/westjem.2015.2.24909 (PMC4427207; doi:10.5811/westjem.2015.2.24909)
Supplement: Supplementary file 1 [file wjem-16-381-s001.pdf]

Project ASSERT Overdose Education Quality Improvement Questionnaire
Ver: 10/13/11

**Interviewer:____________________**	**Date of Interview: ___ ___/ ___ ___/ ___ ___**<br>**MM DD YY**

OVERDOSE EDUCATION AND NARCAN HISTORY

1. How long ago did you visit with Project ASSERT? [Prompt with visit date in IBEX]
   ___ ___ months [convert days and years to months]

2. Have you received overdose education here at Boston Medical Center from Project Assert?
   1☐ Yes
   2☐ No

3. Have you received overdose education elsewhere?
   1☐ Yes
   2☐ No
   b. If yes, from where? ________________________ (Write-in)

4. Have you received a narcan rescue kit from Project Assert?
   1☐ Yes a. When? ___ ___/ ___ ___/ ___ ___ [Confirm the date in the database]
   2☐ No

5. Have you ever received a narcan rescue kit elsewhere?
   1☐ Yes a. When? ___ ___/ ___ ___/ ___ ___
   2☐ No
   11. If yes, from where?
   1☐ Needle exchange
   2☐ Drop-in Center
   3☐ Detox Program
   4☐ From a friend/acquaintance
   5☐ Methadone program
   6☐ Other __________________

   IF NEVER RECEIVED A NARCAN RESCUE KIT, SKIP TO QUESTION #9

6. Where is your narcan rescue kit now?
   1☐ Used by me to reverse an overdose
   2☐ Used by someone else to reverse and overdose
   3☐ With me now
   4☐ Where I live
   5☐ With my friend
   6☐ Lost
   7☐ Other ________________________________

IF THE KIT HAS NOT BEEN USED TO REVERSE AND OVERDOSE, SKIP TO QUESTION #9

7. If since obtaining your narcan rescue kit, you or someone else used it to reverse an overdose, who was it used on?

1☐ Significant other/Spouse	5☐ Friend
2☐ Parent	6☐ Stranger
3☐ Sibling	7☐ Self
4☐ Child	8☐ Other ___________

8. Did it successfully reverse the overdose?

1☐ Yes

2☐ No

PERSONAL OVERDOSE HISTORY

9. How many times have you overdosed since you were at Project ASSERT on ___ ___/ ___ ___/ ___ ___?

____ _____

IF ZERO OVERDOSES, SKIP TO QUESTION #12

Last Overdose

10. When was your last overdose? ___ ___/ ___ ___/ ___ ___?

11. Did you receive narcan?

1☐ Yes, from emergency medical provider

2☐ Yes, from non-medical person a. Was the narcan from your narcan rescue kit? 1☐ Yes 2☐ No

3☐ No

4☐ Don't know/Don't remember

OVERDOSE BYSTANDER HISTORY

12. How many overdoses have you *witnessed* since you were at Project ASSERT on ___ ___/ ___ ___/ ___ ___? _____ _____

IF ZERO OVERDOSES, SKIP TO QUESTION #18

Last Overdose

13. Did the victim receive narcan?

1☐ Yes, from emergency medical provider

2☐ Yes, from me

3☐ Yes, from other non-medical person

4☐ No

5☐ Don't know/Don't remember

a. If yes, how many doses did the victim receive? ___ ___

14. Did you perform rescue breathing?

  1☐ Yes

  2☐ No

  a. Did anyone else perform rescue breathing?

  1☐ Yes

  2☐ No

15. Did you call 911 or ask for emergency medical help?

  1☐ Yes

  2☐ No

  a. Did anyone else call 911 or ask for emergency medical help?

  1☐ Yes

  2☐ No

16. Did you stay with the victim until he or she got better or emergency medical personnel came to help the victim?

  1☐ Yes

  2☐ No

  a. Did anyone else stay with the victim until he or she got better or emergency medical personnel came to help the victim?

  1☐ Yes

  2☐ No

  3☐ Don't know/Don't remember

17. What was your relationship with the person who overdosed the *last time you witnessed an overdose*?

  1☐ Significant other/Spouse   4☐ Child

  2☐ Parent   5☐ Friend

  3☐ Sibling   6☐ Stranger

30 day substance use

18. How many days in the last 30 days have you used (No use in the past 30 days = 00):

Alcohol	____ ____				
Cocaine	____ ____				
Methamphetamine	____ ____				
Marijuana	____ ____				
Heroin	____ ____				
Methadone	____ ____	☐Program	☐Detox	☐Prescribed	☐Not Prescribed
Suboxone	____ ____	☐Program	☐Detox	☐Prescribed	☐Not Prescribed
Percocet, oxycodone, OxyContin, "Perc 30s", "Roxys"	____ ____			☐Prescribed	☐Not Prescribed
Vicodin/ hydrocodone	____ ____			☐Prescribed	☐Not Prescribed
Fentanyl/ Duragesic	____ ____			☐Prescribed	☐Not Prescribed
Other opioid, name the opioid: _______________	____ ____		☐Detox	☐Prescribed	☐Not Prescribed

Benzodiazepines (Klonopin, Xanax, Ativan, Valium, etc.)	____ ____	☐Detox	☐Prescribed	☐Not Prescribed
Clonidine	____ ____	☐Detox	☐Prescribed	☐Not Prescribed
Phenergan	____ ____		☐Prescribed	☐Not Prescribed
Neurontin	____ ____		☐Prescribed	☐Not Prescribed
Seroquel	____ ____		☐Prescribed	☐Not Prescribed
Baclofen	____ ____		☐Prescribed	☐Not Prescribed
Other: _____________	____ ____	☐Detox	☐Prescribed	☐Not Prescribed

30day overdose risk

19. How many days out of the last 30 days have you used opioids (heroin, methadone, suboxone, morphine, oxycodone, Percocet, etc.):
    a. With cocaine, alcohol, benzodiazepines, or other pills, like clonidine, phenergan, seroquel, or neurontin? ____ ____
    b. While you were alone, meaning no one would find you if you overdosed? ____ ____
    c. After 2 days or more of abstinence, like after being incarcerated, after leaving detox, or being hospitalized? ____ ____

Overdose Risk Knowledge Assessment

20. What increases a person's risk for opioid overdose? Please try to think of at least 4 things.
    1☐ Mixing opioids with other psychoactive substances
    2☐ Periods of abstinence
    3☐ Using alone
    4☐ Chronic medical conditions, such as liver, kidney or lung disease
    5☐ Other ____
    [Total the number of correct answers without prompting or clueing the respondent]: ________

General Quality Improvement questions

21. How important do you think it is to learn how to prevent, recognize and respond to an overdose?
    1☐ Very important
    2☐ Somewhat important
    3☐ Not important

22. Is the emergency department or urgent care a good place to receive overdose education?
    1☐ Yes
    2☐ No
    a. Why or Why not?_________________________________ (Write-in)

23. What are the good and bad things about overdose counseling in the Emergency Department?
    ____ (Write-in)
    ____
    ____
    ____

24. How do you think Project ASSERT can improve its efforts at overdose education and naloxone distribution? ____ (Write-in)
____
____
____

<u>Demographics from SDK</u>

25. ED Discharge Diagnosis on day of Project ASSERT visit: __________________________________

26. Gender?
    1☐ Female
    2☐ Male
    3☐ FtM Transgender
    4☐ MtF Transgender

27. Years old? ___ ___

28. Race?
    1☐ African American/Black
    2☐ Asian
    3☐ White
    4☐ Other: ____________________

29. Ethnicity?
    1☐ Hispanic/Latino/Latina
    2☐ Other: ____________________
