# Supplementary material for: Opioid Education and Nasal Naloxone Rescue Kits in the Emergency Department
Source: West J Emerg Med. 2015 Apr 1;16(3):381–4. doi: 10.5811/westjem.2015.2.24909 (PMC4427207; doi:10.5811/westjem.2015.2.24909)
Supplement: Supplementary file 2 [file wjem-16-381-s002.pdf]

Naloxone for Overdose Prevention

patient name

date of birth

patient address

patient city, state, ZIP code

℞

prescriber name

prescriber address

prescriber city, state, ZIP code

prescriber phone number

Naloxone HCl 1 mg/mL
2 x 2 mL as pre-filleld Luer-Lock needless syringe
(NDC 76329-3369-1)

Refills: _____

2 x Intranasal Mucosal Atomizing Device (MAD 300)

Refills: _____

For suspected opioid overdose, spray 1mL in each nostril.
Repeat after 3 minutes if no or minimal response.

Pharmacist: Call 1-800-788-7999 to order MAD 300.

prescriber signature

date

Detach for patient

How to Avoid Overdose

- Only take medicine prescribed to you
- Don't take more than instructed
- Call a doctor if your pain gets worse
- Never mix pain meds with alcohol
- Avoid sleeping pills when taking pain meds
- Dispose of unused medications
- Store your medicine in a secure place
- Learn how to use naloxone
- Teach your family + friends how to respond to an overdose

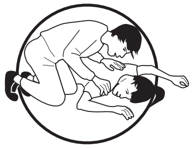

Are they breathing? → Call 911 for help

Signs of an overdose:

- Slow or shallow breathing
- Gasping for air when sleeping or weird snoring
- Pale or bluish skin
- Slow heartbeat, low blood pressure
- Won't wake up or respond (rub knuckles on sternum)

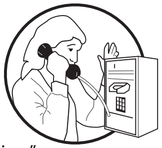

All you have to say:
"Someone is unresponsive and not breathing."
Give clear address and location.

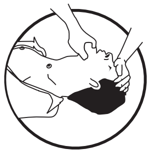

Airway → Rescue breathing

Make sure nothing is inside the person's mouth.

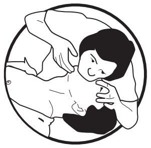

Oxygen saves lives. Breathe for them.
One hand on chin, tilt head back, pinch nose closed.
Make a seal over mouth & breathe in
1 breath every 5 seconds
Chest should rise, not stomach

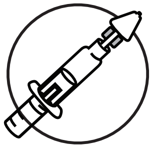

Prepare Naloxone

Are they any better? Can you get naloxone and prepare it quickly enough that they won't go for too long without your breathing assistance?

PrescribeToPrevent.org

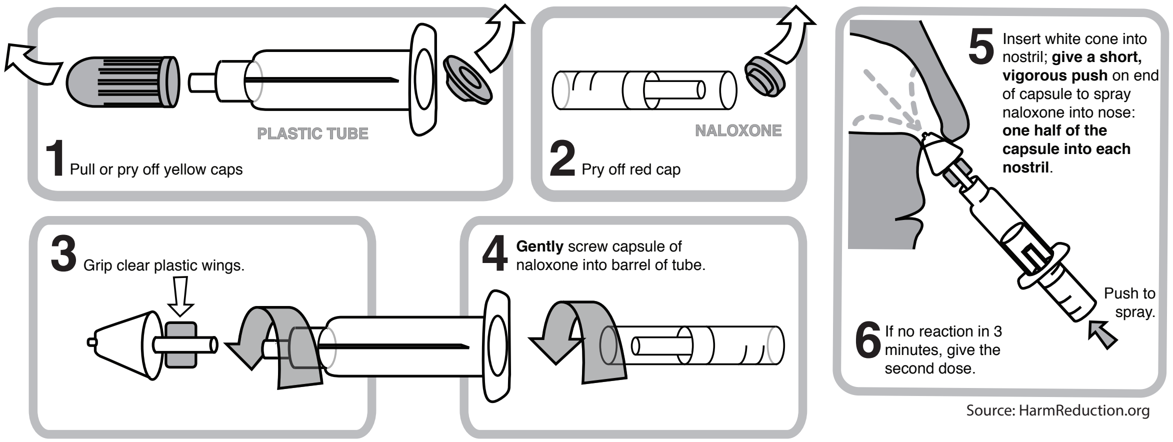

Source: HarmReduction.org

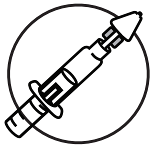

Evaluate + support

- Continue rescue breathing
- Give another 2 sprays of naloxone in 3 minutes if no or minimal breathing or responsiveness
- Naloxone wears off in 30-90 minutes
- Comfort them; withdrawal can be unpleasant
- Get them medical care and help them not use more opiate right away
- Encourage survivors to seek treatment if they feel they have a problem

POISON Help
1-800-222-1222
AAPCC

v02.12.11
